# Gallic acid reduces cell viability, proliferation, invasion and angiogenesis in human cervical cancer cells

**DOI:** 10.3892/ol.2013.1632

**Published:** 2013-10-15

**Authors:** BING ZHAO, MENGCAI HU

**Affiliations:** Department of Health, The Third Affiliated Hospital of Zhengzhou University, Zhengzhou, Henan 450052, P.R. China

**Keywords:** gallic acid, cervical cancer, proliferation, angiogenesis, invasion

## Abstract

Gallic acid is a trihydroxybenzoic acid, also known as 3,4,5-trihydroxybenzoic acid, which is present in plants worldwide, including Chinese medicinal herbs. Gallic acid has been shown to have cytotoxic effects in certain cancer cells, without damaging normal cells. The objective of the present study was to determine whether gallic acid is able to inhibit human cervical cancer cell viability, proliferation and invasion and suppress cervical cancer cell-mediated angiogenesis. Treatment of HeLa and HTB-35 human cancer cells with gallic acid decreased cell viability in a dose-dependent manner. BrdU proliferation and tube formation assays indicated that gallic acid significantly decreased human cervical cancer cell proliferation and tube formation in human umbilical vein endothelial cells, respectively. Additionally, gallic acid decreased HeLa and HTB-35 cell invasion *in vitro*. Western blot analysis demonstrated that the expression of ADAM17, EGFR, p-Akt and p-Erk was suppressed by gallic acid in the HeLa and HTB-35 cell lines. These data indicate that the suppression of ADAM17 and the downregulation of the EGFR, Akt/p-Akt and Erk/p-Erk signaling pathways may contribute to the suppression of cancer progression by Gallic acid. Gallic acid may be a valuable candidate for the treatment of cervical cancer.

## Introduction

Cervical cancer is the second most common type of cancer and the sixth leading cause of cancer-related mortality in females worldwide (1). Cervical cancer affects ~16 females per 100,000 per year and ~8 per 100,000 per year are projected to succumb to the disease (2). Globally, in 2008, it was estimated that there were 473,000 cases of cervical cancer and 253,500 mortalities per year (3,4). Early cervical cancer may be cured by removing or destroying the precancerous or cancerous tissue. However, patient outcome greatly suffers once the cancer has metastasized to distant organs. The standard treatments for cervical cancer include surgery, chemotherapy and radiation therapy. These treatments indiscriminately lead to harm or damage to adjacent or distant normal tissues, and may facilitate cancer cell invasion and metastasis. Cervical cancer metastasis may cause the treatment failure of conventional therapy, including surgery, radiotherapy and chemotherapy (5). Therefore, these studies indicate that new and more effective treatments for cervical cancer require development.

Gallic acid, a naturally occurring plant phenolic compound, is present in a wide variety of plant-based foods. Gallic acid is one of the major active component of Chinese gall, and is widely distributed in natural herbal plants and in large amounts of prescribed Chinese herbs (6,7). In the present study, gallic acid was isolated as a natural antioxidant from Chinese gall. In the past, gallic acid has been reported as a free radical scavenger, able to function to induce differentiation and apoptosis in leukemia and lung cancer and to suppress tumor angiogenesis and cell metastasis. We conjectured that gallic acid plays a significant role in anticancer activities (8–10). Additionally, there are few papers with regard to the effect of gallic acid in cervical cancer cells (11). Therefore, the current study investigated the effect of gallic acid in human cervical cancer HeLa and HTB-35 cells.

## Materials and methods

### Materials

Gallic acid was obtained from Tianjin Yi Fang Ke Ji, Inc. (Tianjin, China). Gallic acid (100 mg) was dissolved in 1 ml dimethyl sulfoxide (DMSO; Shanghai Yanhui Bio-tech Co., Ltd., Shanghai, China) as a stock solution (100 mg/ml). This stock solution was further diluted to varying concentrations (0, 10, 15, 20, 25, 30 and 40 μg/ml) using cell culture medium immediately prior to use. The control group was always treated with the same concentration of DMSO.

### Cell culture

Human cervical cancer HeLa and HTB-35 cells were obtained from the American Type Culture Collection (Rockville, MD, USA) and maintained in DMEM containing 10% fetal bovine serum (FBS; Hangzhou Sijiqing Biology Engineering Materials Co., Ltd., Hangzhou, China), 100 U/ml penicillin, 50 μg/ml streptomycin and 100 μg/ml amphotericin. Cell cultures were were maintained in an incubator with 5% CO_2_ at 37ºC. HUVECs were isolated from fresh human umbilical cord veins and maintained in MEMα (Invitrogen, Carlsbad, CA, USA) supplemented with 20% FBS, 0.04% hydrocortisone, 0.1% VEGF, 0.1% IGF-1, 0.4% hFGF-B, 0.1% hEGF, 0.1% ascorbic acid and 1% heparin.

### MTT (3-(4,5-dimethylthiazol-2-yl)-2,5 diphenyltetrazolium bromide) assay

The MTT assay functions as an indicator of cell survival and/or growth. The assay determines the presence of live cells with functional mitochondria and may be used to detect cytotoxicity and cell growth. Briefly, cervical cancer cells or normal cells were placed into 96-well plates. Subsequent to incubation overnight, the cultured cells were treated with varying concentrations (0, 10, 15, 20, 25, 30 and 40 μg/ml) of gallic acid for 24 h. MTT (Sigma, Shanghai, China) was added to each well and incubated for an additional 4 h at 37ºC. Following centrifugation, the medium containing non-metabilized MTT was then aspirated, and 150 μl DMSO was added to solubilize the formazan. The absorption was then measured at a wavelength of 495 nm by an ELISA plate reader (Varioskan Flash multimode reader, Thermo Fisher Scientific, Shanghai, China).

### Sulforhodamine B (SRB) assay

Skehan *et al* developed the SRB assay in order to measure drug cytotoxicity and cell proliferation for large-scale drug screening. The basis of this assay is the ability of SRB to bind with cellular protein at differing pH values. The optical density reading of the SRB assay is linear with cell number and cellular protein (12). Briefly, the cells were cultured and treated with various concentrations (0, 10, 15, 20, 25, 30 and 40 μg/ml) of gallic acid. Following 24 h of incubation, the cells were fixed with 10% trichloroacetic acid and stained with 0.4% SRB (Sigma) for 30 min. The excess dye was removed by washing repeatedly with 1% acetic acid, then the protein-bound dye was dissolved in 10 mM Tris base solution for examination of the optical density at 510 nm using an ELISA plate reader.

### Bromodeoxyuridine (BrdU) proliferation assay

The BrdU cell proliferation assay is an immunoassay for the quantification of BrdU, which is incorporated into newly synthesized DNA during the proliferative period of the cells. A total of 10,000 cells in 250 μl culture medium were placed into 8-well chambers. The cells were treated with various concentrations (0, 10, 12.5 and 15 μg/ml) of gallic acid, then incubated with BrdU (25 μg/ml; Sigma) and fixed in 4% paraformaldehyde for 30 min. The cells were then incubated with 2N HCl at 37ºC for 10 min. Subsequent to incubation with 0.1 M boric acid for 3 min and being blocked with 1% bovine serum albumin for 1 h, the cells were incubated with anti-BrdU antibody (Millipore, Beijing, China) overnight. Next, the cells were incubated with a FITC-conjugated secondary antibody (Guangzhou Biological Technology Co., Ltd., Guangzhou, China) and then with DAPI (Sigma) at 10 μg/ml for 10 min, prior to being mounted using coverslips. Four random fields from each well were counted under a fluorescent microscope (Nikon, Japan).

### Wound scratch assay

The wound scratch assay is an easy, economical and well-developed method to quantify the migration rate of cells *in vitro*. Brief, an artificial gap was created on the cell monolayers with a plastic pipette tip following treatment with various concentrations (0, 10, 15 and 20 μg/ml) of gallic acid. Images of the wound area were captured following 24 h of incubation under a phase-contrast microscope. Images of three random fields were captured, and the cell migration ability was quantified as the closure of the gap distance.

### Invasion assay

Based on chemoattraction, the Matrigel invasion assay is an extremely fast, low-cost and flexible method to quantify the invasive ability of the majority of cell types. This assay may be used to examine the migration activity associated with matrix degradation. In the present study, Matrigel invasion chambers (BD, Shanghai, China) were used to examine the ability of cervical cancer cells to penetrate the extracellular matrix (ECM). Subsequent to being treated with gallic acid for 24 h, the cells were resuspended in serum-free medium and added to the upper chamber, while medium containing 10% FBS was placed into the lower chamber, thus serving as a chemoattractant. Following incubation for 24 h, the cells on the bottom surface of the base membrane were stained with Cell Tracker Green (Molecular Probes, Eugene, OR, USA) and fixed in 4% formaldehyde for 10 min. Images of four fields were captured randomly and the cells were counted in each field under a fluorescence microscope at ×200 magnification. Data were expressed as the invasive cell number compared with the control. All the experiments were performed in triplicate and the results expressed as the mean ± SEM of three independent experiments.

### Tube formation assay

One of the most popular *in vitro* assays to model the reorganization stage of angiogenesis is the tube formation assay. Usually, this assay is employed to determine the capacity of various compounds to increase or inhibit the formation of capillary-like structures (tube formation). In the present study, 70% ECM gel (100 μl; BD) was added to each well of a 96-well plate, then placed in an incubator at 37ºC to allow the formation of a gel. Subsequent to being treated with various concentrations (0, 5, 10 and 15 μg/ml) of gallic acid, the HUVECs were re-suspended in 150 μl serum-free medium, then placed onto the solidified ECM gel and incubated for 2 h. The endothelial tubes of 5 random fields were examined under a phase-contrast microscope (Nikon), and the extent of tube formation was estimated by counting the overall tube length per area.

### Western blot analysis

Subsequent to being treated with the various concentrations (0, 10, 15 and 20 μg/ml) of gallic acid for 24 h, the HeLa and HTB-35 cells were harvested and rinsed with PBS, followed by extraction in 200 μl RIPA lysis buffer. Equal amounts of each sample were separated by 10% Tris-Glycine gels then transferred to PVDF membranes (Whatman, Hangzhou, China). The membranes were blocked using skimmed milk, followed by incubation with primary antibodies against ADAM17, EGFR, Akt, p-Akt, Erk, p-Erk and actin (Santa Cruz Biotechnology, Dallas, TX, USA). The membranes were analyzed after being incubated with horseradish peroxidase-conjugated secondary antibodies, followed by the use of a SuperSignal West Pico chemiluminescent protein detection kit (Pierce, Rockford, Il, USA).

### Statistical analysis

Data are presented as the mean ± SEM. Statistical significance was analyzed by one-way ANOVA using the GraphPad Prism software (version 4.0; La Jolla, CA, USA). P<0.05 was considered to indicate a statistically significant difference.

## Results

### Gallic acid reduces the viability of cervical cancer cells

**Figure 1:**
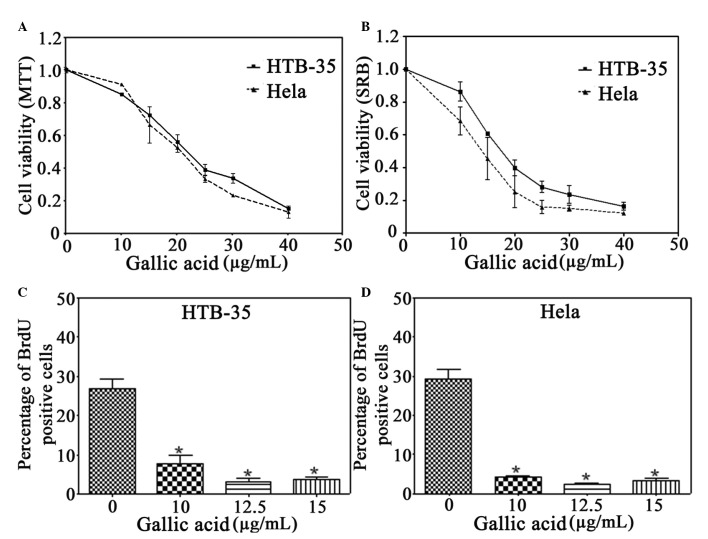
Effect of gallic acid on cell viability and growth of cervical cancer cells. (A) MTT assay of HeLa and HTB-35 cervical cancer cells treated with various concentrations of gallic acid. (B) SRB assay of HeLa and HTB-35 cervical cancer cells treated with the same concentration of MTT. (C) HeLa and HTB-35 cells were treated with the indicated concentration of gallic acid for up to 96 h in complete medium. The MTT assay was used to examine the growth of cells in culture. (D) BrdU incorporation assay of HeLa and HTB-35 cells treated with different doses of gallic acid for 24 h. The cell nuclear incorporation of BrdU was measured. The proliferation rate was presented as the mean ± SEM of the percentage of BrdU-labeled cells vs. DAPI-labeled cells (data obtained from three independent experiments). ^*^P<0.01, control vs. treated groups. MTT, 3-(4,5-dimethylthiazol-2-yl)-2,5-diphenyltetrazolium bromide; BrdU, bromodeoxyuridine; O.D., optical density; SRB, sulforhodamine B.

Gallic acid is an effective chemopreventive agent *in vivo* and *in vitro*(6,7,13). In the first experiment, two major active components, tannic acid and gallic acid, from Chinese gall were compared with its aqueous crude extract. Cell viability was determined by MTT assay using logarithmically growing HeLa and HTB-35 cervical cancer cells treated with various concentrations for 24 h. Tannic acid had no greater cytotoxic effect on the HeLa and HTB-35 cells than the crude extract (data not shown). However, gallic acid significantly reduced the cell viability in a dose-dependent manner (Fig. 1). Therefore, gallic acid was selected for the subsequent experiments. Following treatment with gallic acid for 24 h, the cell viability was significantly decreased in the HeLa and HTB-35 cervical cancer cells, as examined by MTT and SRB assays (Fig. 1A and B). In comparison with the cytotoxic effect on the HeLa and HTB-35 cervical cancer cells, gallic acid exhibited less cytotoxicity in normal HUVECs. Gallic acid reduced cell viability to ~92, 84 and 66% of the control in the HeLa cells and to ~94, 88 and 64% of the control in the HTB-35 cells at concentrations of 5, 10 and 15 μg/ml, respectively. However, at the same concentrations, gallic acid was only able to decrease the cell viability to ~120, 111 and 75% of the control, respectively, in the HUVECs (data not shown). These results provide direct evidence that gallic acid has selective dose-dependent cytotoxicity for cervical cancer cells.

To determine whether gallic acid had a time-dependent effect on the cervical cancer cells, the cells were treated with concentrations of 0, 10, 12.5 or 15 μg/ml, and an MTT assay was performed at 24, 48, 72 and 96 h (Fig. 1C). Gallic acid was able to induce significant inhibition of cell proliferation in the HeLa and HTB-35 cells, but only in a dose-dependent manner. The HeLa cells were more sensitive to gallic acid than the HTB-35 cells at the same concentration of 10 μg (Fig. 1C).

### Gallic acid inhibits proliferation of cervical cancer cells

To elucidate whether gallic acid contributes to the inhibition of cell proliferation, a BrdU incorporation assay was performed on the HeLa and HTB-35 cells treated with 10, 12.5 and 15 μg/ml of gallic acid for 24 h (Fig. 1D). Gallic acid significantly decreased the percentage of BrdU-positive HeLa cells from 27% of the control group to 3.7%. By contrast, the percentage of BrdU-positive HTB-35 cells was reduced from 29% of the control group to 3.3%. These results indicate that gallic acid elicits an antiproliferative effect in cervical cancer cells.

### Gallic acid reduces cervical cancer cell migration and invasion

**Figure 2:**
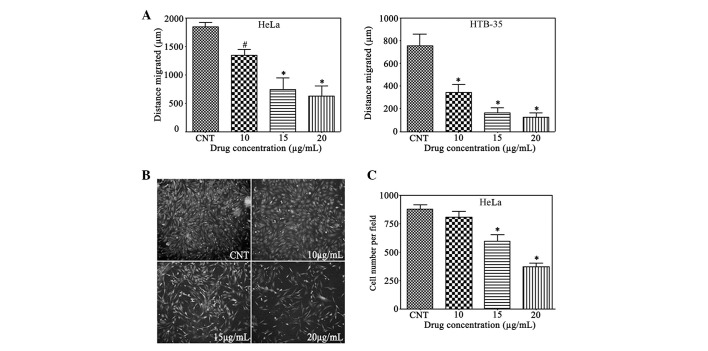
Gallic acid inhibits HeLa and HTB-35 cell migration, and reduces the invasiveness of HeLa cells. (A) In the wound scratch assay, the migration ability was presented as the mean ± SEM of the migration distance. (B) Microscopy images of detected cells that migrated into the lower chamber (magnification, ×200). (C) Cell migration was quantified by the cell number per field. ^#^P<0.05 and ^*^P<0.01, vs. the control (CNT) group.

If untreated, certain cervical high-grade pre-invasive squamous intraepithelial lesions progress to become invasive squamous cell carcinoma and spread to other areas of the body through the blood and lymphocyte system (14). To study the contribution of gallic acid to the cancer cell migration and invasion ability, a wound-scratch assay and Matrigel invasion assay were performed on the cervical cancer cells. In comparison with the untreated group, in the HeLa and HTB-35 cervical cancer cells (Fig. 2A), the closure of the gap distance was inhibited significantly and dose-dependently by gallic acid at 10, 15, and 20 μg/ml. Gallic acid displayed differing inhibitory effects on the different cell lines. In comparison with the HeLa cells, gallic acid decreased the gap to a greater extent at the same concentrations in the HTB-35 cells. At concentrations of 10, 15, and 20 μg/ml, gallic acid was able to dramatically inhibit cell migration to 73, 40 and 34% in the HeLa cells and to 45, 22 and 17% in the HTB-35 cells, respectively, compared with the control. Invasiveness is an important characteristic of cervical cells and a target for the development of anticancer agents (14). As shown in Fig. 2B and C, gallic acid significantly reduced the invasiveness of the HeLa cells (P<0.05) to ~92, 68 and 29% of the control at the concentrations of 10, 15, and 20 μg/ml.

### Gallic acid inhibits angiogenesis

**Figure 3:**
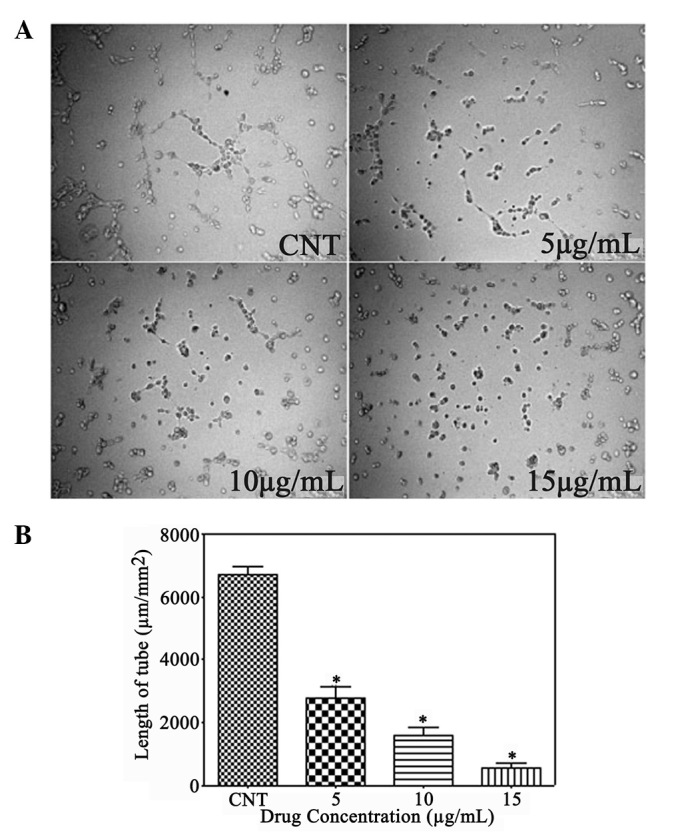
Effect of gallic acid on human umbilical vein endothelial cell (HUVEC) tubulogenesis *in vitro*. (A) Representative photomicrographs during the tube formation of HUVECs pretreated with 5, 10 or 15 μg/ml gallic acid for 24 h. (B) The ability to form tubes was expressed as ratios of the length of formed tubes per picture field. ^*^P<0.01, vs. the control (CNT) group.

Angiogenesis is the formation of new blood vessels, which is considered a critical step for the growth of solid tumors. Due to the neovascular nature of cervical cancer (15,16), the present study investigated whether gallic acid had the ability to inhibit tube formation in HUVECs. The untreated control group was composed of multiple cells that gathered together and adhered to each other. However, gallic acid showed significant inhibition of the elongation of the tubes at all concentrations, and the tube length per area was decreased to ~16.5, 15.3 and 30.3% of the control group, respectively (Fig. 3A and B).

### Gallic acid suppresses ADAM17 and EGFR expression in cervical cancer cells

**Figure 4:**
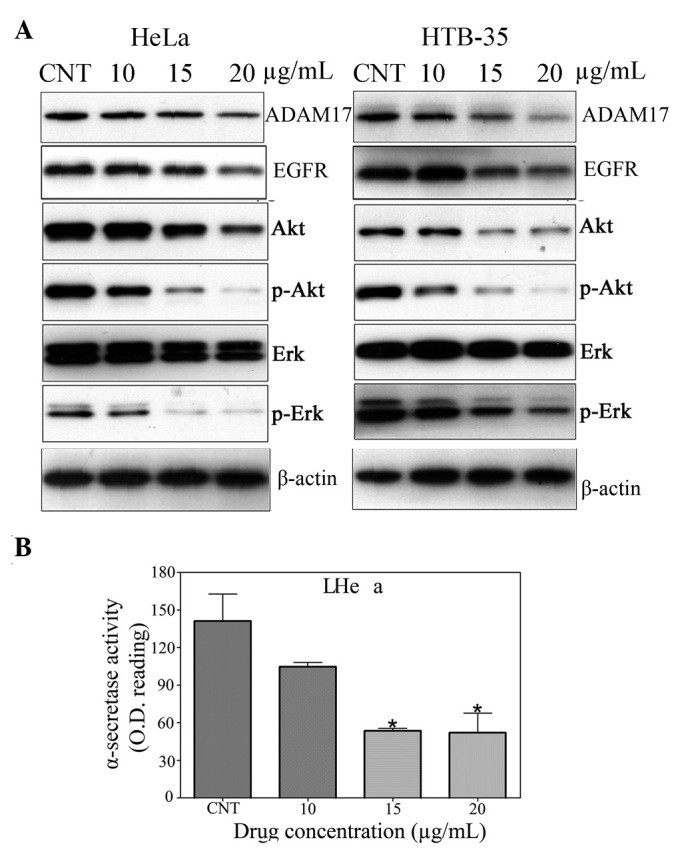
(A) Western blot analysis of the expression of ADAM17, EGFR, Erk/p-Erk and Akt/p-Akt in HeLa and HTB-35 cells. The cells were treated with 10, 15 or 20 μg/ml gallic acid for 24 h, and subjected to immunoblotting with antibodies against ADAM17, EGFR, Erk/p-Erk and Akt/p-Akt. β-actin was used as a sample loading control. (B) Effect of gallic acid on ADAM17 α-secretase activity in HeLa cells. ^*^P<0.01, vs. the control (CNT) cells. O.D., optical density.

It has been reported that ADAM17 contributes to cancer cell progression through activation of the EGFR/PI3K/Akt and EGFR/Ras/MAPK/Erk signaling pathways (17,18). It is evident that EGFR is expressed in normal and cervical cancers with varying degrees of EGFR expression (19–21). Therefore, these make cervical cancer amenable for targeted therapy. EGFR expression acts as a biomarker for forming a prognosis or for administering treatment (22). To elucidate whether gallic acid was able to inhibit cervical cancer cell invasion by decreasing the levels of ADAM17 and EGFR, the expression of ADAM17, Erk/p-Erk and Akt/p-Akt was examined by western blotting in the HeLa and HTB-35 cervical cancer cells following treatment with gallic acid (10, 15, 20 μg/ml) for 24 h. In each cell line, the expression of ADAM17, EGFR, p-Erk and p-Akt was significantly inhibited by gallic acid (Fig. 4A). To further verify the effect of gallic acid on ADAM17, an α-secretase activity assay kit was employed to measure ADAM17 activity. Subsequent to being treated with gallic acid at 20, 30, and 40 μg/ml for 24 h, ADAM17 activity was significantly decreased to approximately 74.7 (P<0.05), 38.8 (P<0.01) and 37.6% (P<0.01) of the control group, respectively (Fig. 4B). These results indicate that gallic acid elicits a reduction in tumor invasiveness through the downregulation of ADAM17 and EGFR expression and the dephosphorylation of Erk and Akt.

## Discussion

The present study examined the effect of gallic acid, a major active component of Chinese gall, which is a traditional Chinese medical herb that has been used to treat various cancers. The results of the MTT and SRB assays showed that gallic acid significantly decreased the cell viability of the HeLa and HTB-35 cancer cells in a dose-dependent manner. In comparison with the effect on the cervical cancer cells, gallic acid showed a substantial reduction of the cytotoxic effects on normal HUVECs at the same concentrations (data not shown). These results indicated that gallic acid exhibits significantly selective cytotoxicity in cervical cancer. This data therefore demonstrates that gallic acid reduces cervical cancer cell proliferation. The HeLa and HTB-35 cells were severely affected by gallic acid in the BrdU incorporation assay. Previous studies have reported that gallic acid has the ability to induce apoptosis in esophageal cancer (6), human prostate cancer (13) and HL-60 promyelocytic leukemia (23) cells. However, there was no significant apoptosis observed in either cell line following the employment of Hoechst 33342 staining and a TUNEL apoptosis assay in the pilot study.

The vascular network provides all the cells in the body with oxygen and nutrients. It is evident that oxygen possesses the most significant role in the regulation of solid tumor growth. Due to aberrant growth and the subsequent imbalance of the supply and demand of oxygen, the solid tumors, including those of cervical cancer cells, cause hypoxia, which promotes the growth of new capillaries to acquire more oxygen (16,22,24). Previous studies have reported that gallic acid may be responsible for the decrease of angiogenesis *in vitro* and *in vivo*(25). With the employment of a human placental vein angiogenesis model, gallic acid has been shown to significantly inhibit the initiation of angiogenesis and neovessel growth (25). Additionally, following the intraperitoneal administration of 250 mg *Rubus* extract for 2 days, serum from the rats also exhibited significant inhibition of angiogenic initiation and subsequent neovessel growth. However, subsequent to being gavaged with the extract, serum from the rats did not significantly reduce angiogenic initiation and neovessel growth. Therefore, in the present study, an *in vitro* tube formation assay was performed to examine whether gallic acid has anti-angiogenesis abilities. The HUVECs were less sensitive to the cytotoxicity induced by gallic acid compared with HeLa and HTB-35 cells at the concentrations of 5, 10 and 15 μg/ml. However, at all three concentrations, gallic acid significantly decreased the capillary tube formation in the HUVECs. Therefore, based on this data, gallic acid may be considered useful for targeting angiogenesis. However, further clarification of the underlying mechanisms is necessary.

In the present study, a wound healing assay was employed to investigate the migration of the HeLa and HTB-35 cells, and the Matrigel invasion assay was performed to evaluate the capacity of the HeLa cells to degrade the ECM and migrate through the pores in the membrane. These data showed that the migration of the HeLa and HTB-35 cells was significantly reduced with the treatment of gallic acid. Also, the reduction of the invasion potential by gallic acid was observed in the HeLa cells. To elucidate the underlying mechanisms of decreased invasiveness, the present study analyzed the protein expression of ADAM17 and EGFR and the dephosphorylation state of Erk and Akt with the use of western blotting in the HeLa and HTB-35 cells. Gallic acid significantly reduced the level of ADAM17 and EGFR expression and the level of Akt and Erk phosphorylation in the two cell lines. In addition to the inhibition of protein expression, gallic acid significantly reduced ADAM17 activity at all indicated concentrations.

EGFR is a 170-kDa transmembrane glycoprotein receptor encoded by the Her-1 proto-oncogene located on chromosome 7p12. EGFR functions through dimerization, which activates a tyrosine kinase domain to regulate multiple functions, including cell growth, differentiation, gene expression and development (26). EGFR is present in numerous normal tissues and is expressed in a wide variety of solid tumors, including cervical cancer (27). ADAMs are best known as ectodomain sheddases, and their domains function as metalloproteases (28,29). The disintegrin metalloproteinases of the ADAM family are associated with the process of proteolytic ‘shedding’ of membrane-associated proteins and hence the rapid modulation of key cell signaling pathways in the tumor microenvironment. ADAM17 is an important member of the ADAM family involved in the proteolysis of collagen IV of the ECM and also the release of several integrins from the cell surface, indicating that ADAM17 affects the migration activity of a variety of cells, including cervical cancer cells. ADAM17 is a primary upstream component for multiple EGFR pro-ligands (30). EGFR binds with its ligands and subsequently activates downstream MAPK/ERK and PI3K/Akt pathways, which contribute to invasiveness and other malignant phenotypes (17,18,30). Gallic acid significantly decreases the phosphorylation of members of the PI3K/AKT and MAPK/ERK signaling pathways, which play key roles in cell proliferation and invasion. The present results indicated that the inhibition of ADAM17 by gallic acid may be responsible for decreased invasiveness through the suppression of the EGFR/PI3K/AKT and EGFR/MAPK/ERK pathways.

In summary, the present study demonstrated that gallic acid significantly reduces cell viability, proliferation, invasion and tube formation. Suppression of ADAM17 and EGFR may contribute to the inhibition of invasiveness through the inactivation of PI3K/AKT and MAPK/ERK signaling pathways. These results demonstrate the importance of validating the use of traditional Chinese medicinal herbs in tumor prevention and therapy, and show that gallic acid may potentially serve as a candidate for cervical cancer treatment.
